# The Teeth

**Published:** 1860-07

**Authors:** 


### THE TEETH.

Said Dr. Ostrander, (at the head of his profession in his own State:) "If dentistry had reached its present perfection when I was a young man, the whole tenor of my life would have been altered."

Why?

"I was addressing a young lady of great moral worth, of unusual personal attractions, and the heiress of a large fortune. She had not reached her twentieth year. In a state of repose, her face was perfectly beautiful. But when she smiled, a set of teeth were presented, so discolored, so uneven, so defective and decayed, and the breath was so offensive, that I could not possibly reconcile it to myself to be linked for life to circumstances so repulsive. The very thought of it was abhorrent to me, so I gradually withdrew my attentions, and wedded poverty with a sweet mouth."

Charity may cover a multitude of sins; and a great estate may veil as great a multitude of personal defects, to the uneducated and the vulgar, but the wealth of Crœsus could not reconcile a man of culture and refinement to wed a snaggled tooth and an odoriferous breath. In the matter of lovability, nothing can compensate for the absence of beautiful teeth and a sweet breath. Hence, parents will perform towards their children most important service by doing what they may to secure to them perfectly sound teeth, not only as an important means of preserving health, but as an invaluable aid in forming desirable alliances.

Two things are indispensable: First, from the age of four years, until marriage, have a good dentist to examine every tooth most minutely, several times a year; second, begin quite as early to impress the child with the importance of keeping the teeth clean, and how best to do it.

A child has ten teeth in each jaw; all these, and these only, are shed; generally, in healthy children, two teeth are shown at eight months, at least eight in fourteen months, and the whole twenty at two and a half years.

From five to six years of age the first permanent teeth appear; and from that time the frequent and vigilant services of a sharp-eyed dentist ought to be secured. The eye-teeth ap pear between the eleventh and twelfth year; at fourteen the large double-teeth present themselves, and the wisdom teeth at about twenty.

Hot and cold drinks should be avoided, particularly at the same meal.

The teeth should not be washed in cold water, especially after eating, because the contrast between it and warm or hot food is too striking, and chills them.

Each person should have two tooth-brushes, one moderately stiff, to be employed the first thing in the morning; the other, which may be a morning one, which has been used for some time, should be softer, and should not be used in rubbing across the teeth much, lest it might cause the gums to recede, and thus pave the way for their falling out, but should be twisted up and down, so that each bristle may act as a tooth-pick, to dislodge any particles between the teeth.

These softer brushes should be used immediately after each meal, taking care, at the end of the operation, to pass the brush across the back part of the tongue, and then gargle the mouth and throat well with water.

For cleaning the teeth and mouth, warm water, always at hand in cities, should be used, but never employ water so hot or cold as to cause uncomfortableness to the teeth, for they will soon be destroyed thereby. When it is very inconvenient to have warm water, hold the cold water in the back part of the mouth, keeping it from the teeth with the tongue as much as possible, until it is warmer, and then use the brush.

It is frequently advised to clean the teeth the last thing at night; a much better plan is to do it the first thing after supper, and then they are in a clean condition for four or five hours longer out of every twenty-four, while the trouble of cleaning the teeth a second time would tend to prevent eating any thing later than supper.

The tooth-brush should be always used leisurely, for a slip or inadvertence may scale or break off a valuable tooth. Once or twice a week, the first or last brushing should be with pure white soap, thus: Wet the brush, and draw it several times across the soap, then put it in the mouth, rubbing the teeth until the mouth is full of foam, and for a minute or two employ the brush on the side of the teeth next the tongue, above and below, for it is there that tartar collects, to the eating away of the gums, and eventual falling out of the teeth. In most cases this tartar is deposited by a living creature, which is instantly destroyed by soap-suds, when tobacco-juice and the strongest acids have no effect.

Charcoal, even when made of the bark of wood, is one of the most destructive of all tooth-powders. Eminent dentists agree in this; it finds its way between the teeth and the gums, and destroys both.

Almost all the tooth-powders have a strong acid of some kind, and this cleanses the teeth, but destroys their texture; this may be obviated to a great extent if, immediately after using any tooth-powder, the teeth are well brushed with soap, to antagonize any acid which may be left about them.

If the brush is used as above, powders will not be necessary more than two or three times a year; in our own case, common salt, once in two or three months, seems to have answered an excellent purpose; put on a damp brush, rubbed across and up and down the teeth. It is not advised to keep the teeth always of a pearly whiteness, for they may be cleaned so much as to be worn away. It would be a good plan for a dentist, once a year, to go over every tooth with powdered pummice-stone and a piece of soft wood. Bad teeth induce dyspepsia, from insufficient chewing of the food; they also corrupt the breath, and are frequently the causes of serious and distressing disease; while good teeth not only beautify the face, but promote health and long life; hence, special care expended on their preservation will be repaid an hundred fold in the course of a life-time.

